# Association between sociodemographic status and cardiovascular risk factors burden in community populations: implication for reducing cardiovascular disease burden

**DOI:** 10.1186/s12889-022-14374-4

**Published:** 2022-10-31

**Authors:** Anping Cai, Zhiqiang Nie, Yanqiu Ou, Jiabin Wang, Yanshuang Chen, Zhisheng Lu, Yanhua Liang, Yingling Zhou, Yingqing Feng

**Affiliations:** 1 Department of Cardiology, Hypertension Research Laboratory, Guangdong Cardiovascular Institute, Guangdong Provincial People’s Hospital, Guangdong Academy of Medical Sciences, 510080 Guangzhou, China; 2 Department of Epidemiology, Global Health Research Center, Guangdong Provincial People’s Hospital, Guangdong Academy of Medical Sciences, 510080 Guangzhou, China; 3 Community Health Center of Liaobu County, Dongguan, Guangdong China; 4 Community Health Center of Xiaolan County, Zhongshan, Guangdong China; 5 Center for Disease Control and Prevention of Jiangmen, Jiangmen, Guangdong China; 6 Guangdong Provincial People’s Hospital, Guangdong Academy of Medical Sciences, No.106, Zhongshan 2nd Road, 510080 Yuexiu District, Guangzhou China

**Keywords:** Epidemiology, Risk factor, Cardiovascular disease, Sociodemographic status

## Abstract

**Background:**

We aimed to evaluate the burden of cardiovascular (CV) risk factors in the community populations of Guangdong Province and its association with sociodemographic status (SDS).

**Method:**

The data were from the community populations of Guangdong Province who have participated in the China PEACE Million Persons Project between 2016 and 2020 (n = 102,358, women 60.5% and mean age 54.3 years). The prevalence of CV risk factors (smoking, drinking, overweight/obesity, hypertension, dyslipidemia and diabetes mellitus) and its association with SDS (age, sex and socioeconomic status [SES]) was evaluated cross-sectionally.

**Results:**

The prevalence of overweight/obesity was 48.9%, hypertension 39.9%, dyslipidemia 18.6%, smoking 17.2%, diabetes mellitus 16.1% and drinking 5.3%. Even in young adults (aged 35–44), nearly 60% had at least 1 CV risk factor. Overweight/obesity often coexisted with other risk factors, including smoking, hypertension, dyslipidemia and diabetes mellitus. The proportion of people with no risk factor decreased with increasing age. Women were more likely than men to have no CV risk factor (29.4% vs. 12.7%). People with ≥ high school degree were more likely than those with < high school to have no risk factor (28.5% vs. 20.4%), and farmers were less likely than non-farmers to have no risk factor (20.8% vs. 23.1%).

**Conclusion:**

The burden of CV risk factors is high and varied by SDS in the community populations of Guangdong Province. Cost-effective and targeted interventions are needed to reduce the burden of CV risk factors at the population level.

## Background

The burden of cardiovascular disease (CVD) in China has been consistently increasing since 2006 [1]. CVD causes substantial health and economic burden in China [2, 3]. In 2016, nearly 13% of admission was due to CVD, and the cost for three main CVD (acute myocardial infarction, intracranial hemorrhage and cerebral infarction) was more than 100 billion RMB [1]. Nearly 40% of death was due to CVD in the Chinese adults [4]. Recognizing these burdens, the Chinese government has launched several national health-promotion plans and programs [5]. For example, in 2016, the Chinese government endorsed the blueprint of “Healthy China 2030”, which has made population health the ultimate goal of economic development and political reform. Two of the goals are to improve longevity and healthy life expectancy and to increase disease prevention, aiming at reaching a health standard on par with developed countries by 2030 [6]. To achieve these goals, reducing the CVD burden is imperative.

The burden of CVD can be reduced through effectively preventing and controlling cardiovascular (CV) risk factors [7]. Smoking, drinking, overweight/obesity, hypertension, dyslipidemia and diabetes mellitus are the most common and important CV risk factors. Epidemiologic studies have shown that there was a trend toward increasing the prevalence of CV risk factors in China. For example, the 2018 China Hypertension Survey shows that nearly 23.2% (244.5 million) of adults aged ≥ 18 years had hypertension [8], which was 5.2% higher than that in the 2002 survey [9]. The prevalence of diabetes mellitus increased from 10.9% to 2013 to 12.4% in 2018 [10], and obesity increased in an alarming rate, from 31.9% to 2007 to 37.2% in 2017 [11]. Several studies, that have reported the nationwide status of smoking [12], drinking [12], overweight/obesity [13, 14], hypertension [8, 15], dyslipidemia [16–18] and diabetes mellitus [19, 20], uniformly show that the epidemiological characteristics of CV risk factors differed widely across different regions of China, which might be partly explained by the regional differences in sociodemographic status (SDS).

In recent four decades, Guangdong Province has experienced rapid economic, social, and cultural changes, that might have impacted the epidemiological characteristics of CV risk factors.

Enhancing our knowledge in these aspects is crucial for CVD prevention and management. However, there are no contemporary epidemiological data on the CV risk factors in Guangdong Province. From 2016 to 2020, Guangdong Province has participated in the China Patient-Centered Evaluative Assessment of Cardiac Events (PEACE) Million Persons Project, which is a government-funded, nationwide, and population-based CVD screening study [21, 22]. Leveraging this study, we aimed to (1) evaluate the contemporary CV risk factors burden in Guangdong Province, and (2) evaluate the association between SDS and CV risk factors burden.

## Methods

### Study design and participant

This is a cross-sectional study that followed the Strengthening the Reporting of Observational Studies in Epidemiology (STROBE) reporting guidelines [23]. The current study was approved by both the Central Ethics Committee at the China National Center for Cardiovascular Disease and the Ethics Committee of Guangdong Provincial People’s Hospital (No. GDREC2016438H (R2)). A purposive sampling method was used to sample and select participants [21, 22]. From 2016 to 2020, 8 sites, most were from the developed cities of Guangdong Province, were selected. At each site, participants who were aged 35 to 75 years and currently registered in the selected region’s Hukou (an official record identifying residents in the region) were eligible for this study and were recruited by local staff via extensive publicity campaigns on television and in newspapers after written inform consent was obtained. All the performances were in accordance with the Declaration of Helsinki.

### Data collection and variables

Information on demographics (age and sex), socioeconomic status (SES), smoking and drinking status, comorbid condition, medical history and current medication used was collected during in-person interviews by trained staff. Height and body weight were measured using standard protocols, and body mass index (BMI) was calculated by dividing the weight in kilograms by the square of height in meters. Blood pressure was measured two times on the right upper arm after 5 min of rest in a seated position using an electronic blood pressure monitor (Omron HEM-7430; Omron Corporation, Kyoto, Japan). Fasting plasma glucose (BeneCheck BK6–20 M Multi­Monitoring System, Suzhou Pu Chun Tang Biotechnology, China) and lipid profiles (CardioChek PA Analyzer; Polymer Technology Systems, Indianapolis, Indiana, USA) was measured using fingertip blood samples.

### Cardiovascular risk factor and score

Smoking and drinking status were based on self-report. For example, during the face-to-face interview, participants were asked how often they drank alcohol in the last year, and if he/she answered never then the status was no, otherwise the status was yes, regardless of the frequency of drinking. Based on recommendations of the Working Group on Obesity in China [24], overweight and obesity was defined as BMI ≥ 24 kg/m^2^ and ≥ 28 kg/m^2^, respectively. Besides self-report, hypertension was also defined based on the use of antihypertensive drug, systolic blood pressure ≥ 140 mm Hg or diastolic blood pressure ≥ 90 mm Hg; dyslipidemia was defined based on the use of lipid-lowering medication, total cholesterol ≥ 6.2 mmol/L or low-density lipoprotein cholesterol ≥ 4.1 mmol/L; and diabetes mellitus was defined based on the use of antidiabetic drug, or fasting plasma glucose ≥ 7.0 mmol/L. Each risk factor was assigned one score, and the score ranged from 0 to 6. A higher score indicated a larger CV risk factor burden.

### Statistical analysis

In this study, participants were divided into several subgroups based on age (35–44, 45–54, 55–64 and 65–75 years), sex, education attainment (< high school and ≥ high school), annual household income (< 50,000 and ≥ 50,000 RMB), residency urbanity (rural area and urban), and occupation (farmer and non-farmer), respectively. Differences in CV risk factors were evaluated according to these subgroups. Continuous variables were presented as mean and standard deviation (SD), the student t test or U test was used to analyze continuous variables depending on the normality of the data distribution. Categorical variables were presented as frequency (proportion) and were compared by chi-squared test. Cross-tabulation was used to calculate the combination of individual CV risk factor. A multivariable logistic regression analysis was used to evaluate the association between age, sex and SES with CV risk factors, with adjustment for age, sex, education, annual household income, residence urbanity, health insurance status, occupation and marital status. A bootstrap method (1000 resamples) was used to provide the estimates of 95% confidence interval (CI) of the prevalence of CV risk factors. All analyses were conducted with R statistical software version 3.33 (R Project for Statistical Computing). All statistical testing was 2-sided, at a significance level of P-value < 0.05.

## Results

### General characteristics of study participants

A total of 102,358 participants were recruited from 2016 to 2020. The mean age of the study cohort was 54.3 years and women were 60.5% (Table 1). Of study participants, 29.6% had ≥ high school degree, 45.4% had annual household income ≥ 50,000 RMB, 48.4% were from urban, and 11.8% were farmers. The prevalence of smoking was 17.2% (95% CI 16.9–17.5%), drinking 5.3% (95% CI 5.2–5.4%), overweight/obesity 48.9% (95% CI 48.4–49.3%), hypertension 39.9% (95% CI 39.5–40.3%), dyslipidemia 18.6% (95% CI 18.3–18.9%) and diabetes mellitus 16.1% (95% CI 15.8–16.3%). The proportion of those with hypertension, dyslipidemia and diabetes mellitus receiving pharmacological therapy was 48.7%, 20.8% and 42.9%, respectively.

**Table 1:** Characteristics of Study Participants (n = 102,358)

Variables	
Age (years)	54.3 ± 10.2
Age, n (%)	
35–44 years	20,514 (20.0)
45–54 years	32,388 (31.6)
55–64 years	29,949 (29.3)
65–75 years	19,507 (19.1)
Sex, n (%)	
Men	40,440 (39.5)
Women	61,918 (60.5)
Education, n (%)	
< High school	72,063 (70.4)
≥ High school	30,295 (29.6)
Annual household income (RMB), n (%)	
< 50 000	55,938 (54.6)
≥ 50 000	46,420 (45.4)
Health insurance status, n (%)	
Uninsured	6893 (6.7)
Insured	95,465 (93.3)
Residence urbanity, n (%)	
Rural	52,849 (51.6)
Urban	49,509 (48.4)
Marital status, n (%)	
Married	92,599 (90.5)
Others	9759 (9.5)
Occupation, n (%)	
Farmer	12,054 (11.8)
Non-farmer	90,304 (88.2)
Current smoking, n (%)	
No	84,758 (82.8)
Yes	17,600 (17.2)
Current drinking, n (%)	
No	96,932 (94.7)
Yes	5426 (5.3)
Body mass index (kg/m^2^)	24.1 ± 3.3
Overweight/obesity, n (%)	50,016 (48.9)
Systolic blood pressure (mm Hg)	130.1 ± 19.0
Diastolic blood pressure (mm Hg)	79.2 ± 11.3
Hypertension, n (%)	40,866 (39.9)
Dyslipidemia, n (%)	19,044 (18.6)
Diabetes mellitus, n (%)	16,434 (16.1)
TC (mmol/L)	4.91 ± 1.23
LDL-C (mmol/L)	2.72 ± 1.00
HDL-C (mmol/L)	1.48 ± 0.44
TG (mmol/L)	1.35 (0.99–1.94)
Fasting plasma glucose (mmol/L)	5.89 ± 1.68
Myocardial infarction, n (%)	367 (0.4)
Stroke, n (%)	630 (0.6)

TC, total cholesterol; LDL-C, low density lipoprotein-cholesterol; HDL-C, high density lipoprotein-cholesterol; TG, triglyceride

### Prevalence of CV risk factor according to age, sex and SES

**Fig. 1:**
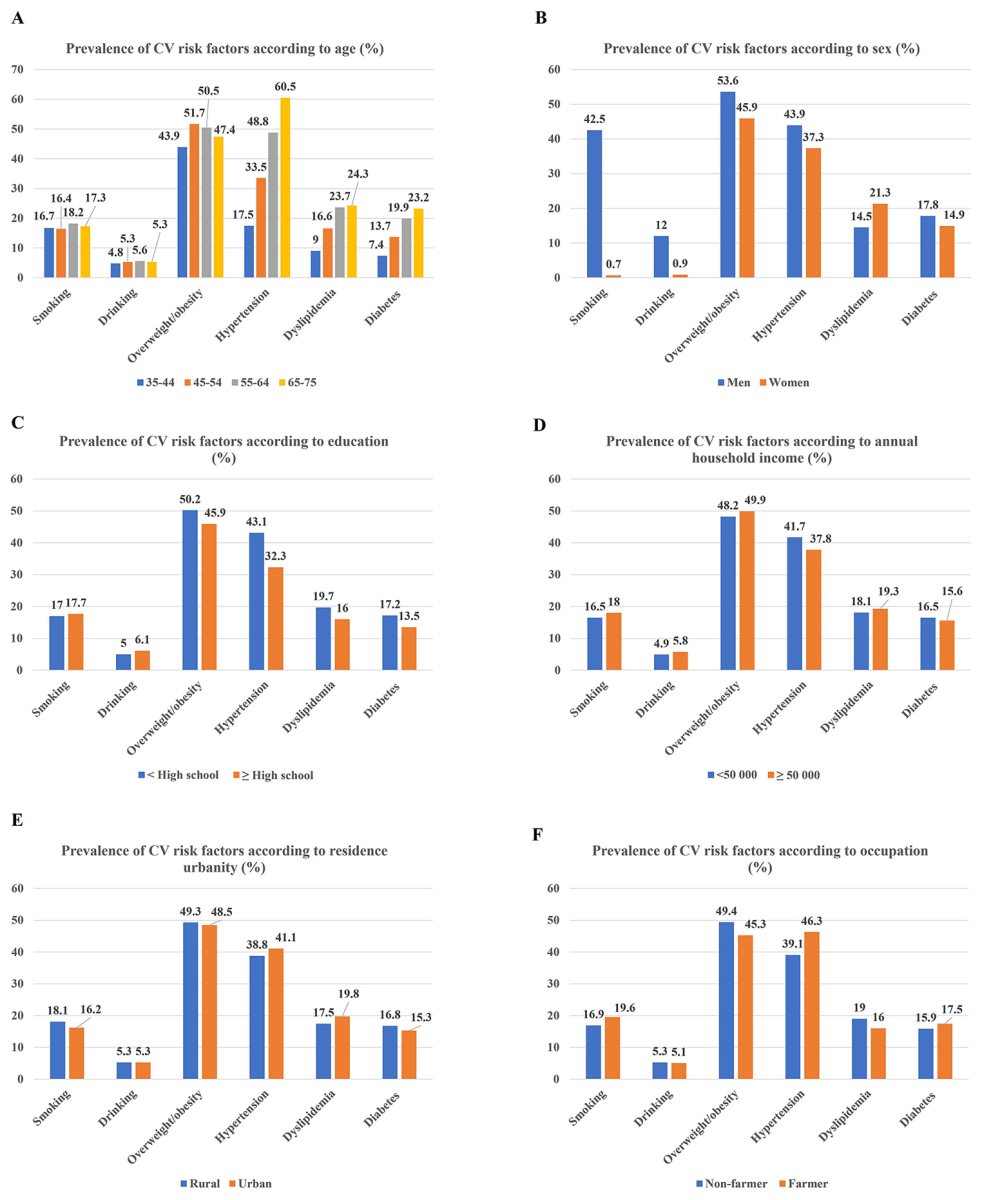
Prevalence of cardiovascular risk factors according to sociodemographic status. Panel A: Prevalence of cardiovascular risk factor according to age. People in the 55–64 age group were more likely to smoke and use alcohol; people in the 45–54 age group had a higher prevalence of overweight/obesity; and people in the 65–75 age group had a higher prevalence of hypertension, dyslipidemia and diabetes mellitus. Panel B: Prevalence of cardiovascular risk factor according to sex. Women were less likely to have these risk factors than men, except that they had a higher prevalence of dyslipidemia. Panel C-F: Prevalence of cardiovascular risk factor according to socioeconomic status. People with ≥ high school degree had a lower prevalence of overweight/obesity, hypertension, dyslipidemia and diabetes mellitus, while they were more likely to smoke and use alcohol. People with annual household income ≥ 50,000 RMB were more likely to smoke and use alcohol and had a higher prevalence of overweight/obesity and dyslipidemia, while they had a lower prevalence of hypertension and diabetes mellitus. People from urban were less likely to smoke and had a lower prevalence of overweight/obesity and diabetes mellitus, while they had a higher prevalence of hypertension and dyslipidemia. Farmer were more likely to smoke and had a higher prevalence of hypertension and diabetes mellitus, while they had a lower prevalence of overweight/obesity and dyslipidemia

The prevalence of CV risk factor according to the age, sex and SES subgroups were summarized in Fig. 1. As shown in Table 2, the mean number of CV risk factors increased with increasing age (35–44 years 0.99 ± 1.03 vs. 45–54 years 1.37 ± 1.12 vs. 55–64 years 1.67 ± 1.13 vs. 65–75 years 1.78 ± 1.1 3; P < 0.001); men had higher mean number of CV risk factors than women (1.84 ± 1.19 vs. 1.21 ± 1.04; P < 0.001); people with < high school degree had higher mean number of CV risk factors than those with ≥ high school (1.52 ± 1.13 vs. 1.32 ± 1.15; P < 0.001); and farmers had higher mean number of CV risk factors than non-farmers (1.50 ± 1.12 vs. 1.46 ± 1.15; P < 0.001). There was no statistically significant difference in the mean number of CV risk factors according to annual household income (< 50,000 RMB 1.46 ± 1.13 vs. ≥ 50,000 RMB 1.46 ± 1.15; P = 0.92) and residence urbanity (Rural area 1.46 ± 1.14 vs. Urban 1.46 ± 1.14; P = 0.37).

**Table 2:** Number of Cardiovascular Risk Factors according to Age, Sex and Socioeconomic Status

Variables	Number of CV Risk Factors
Age	
35–44 years	0.99 ± 1.03
45–54 years	1.37 ± 1.12
55–64 years	1.67 ± 1.13
65–75 years	1.78 ± 1.13
* P*-value	< 0.001
Sex	
Men	1.84 ± 1.19
Women	1.21 ± 1.04
* P*-value	< 0.001
Education	
< High school	1.52 ± 1.13
≥ High school	1.32 ± 1.15
* P*-value	< 0.001
Annual household income (RMB), n (%)	
< 50 000	1.46 ± 1.13
≥ 50 000	1.46 ± 1.15
* P*-value	0.920
Residence urbanity, n (%)	
Rural	1.46 ± 1.14
Urban	1.46 ± 1.14
* P*-value	0.371
Occupation, n (%)	
Farmer	1.50 ± 1.12
Non-farmer	1.46 ± 1.15
* P*-value	< 0.001

CV, cardiovascular

### Association between age, sex and SES with CV risk factors

Unadjusted association between age, sex and SES and CV risk factors was shown in Table 3. After adjusting for covariates (Table 4), increasing age was associated with higher odds of overweight/obesity, hypertension, dyslipidemia and diabetes mellitus. Female sex was associated with lower odds of these risk factors except associated with higher odds of dyslipidemia. Higher education attainment was associated with lower odds of these risk factors except for no association with dyslipidemia. Higher annual household income was associated with higher odds of drinking, overweight/obesity and dyslipidemia. Residence in the urban was associated with lower odds of smoking and diabetes mellitus while associated with higher odds of hypertension and dyslipidemia. There was no association between occupation and CV risk factors.

**Table 3:** Association between Age, Sex and Socioeconomic Status with Cardiovascular Risk Factor

	Smoking	Drinking	Overweight/obesity	Hypertension	Dyslipidemia	Diabetes
	**Unadjusted Odds ratio (95% confidence interval)**					
**Age (years)**						
35–44	Reference					
45–54	0.98 (0.93–1.02)	1.11 (1.03–1.21)	1.37 (1.32–1.41)	2.37 (2.27–2.48)	2.01 (1.90–2.13)	1.99 (1.87–2.11)
55–64	1.11 (1.06–1.16)	1.16 (1.07–1.26)	1.30 (1.26–1.35)	4.49 (4.30–4.68)	3.14 (2.97–3.32)	3.13 (2.94–3.32)
65–75	1.04 (0.99–1.10)	1.11 (1.01–1.21)	1.15 (1.11–1.20)	7.21 (6.88–7.54)	3.25 (3.07–3.44)	3.79 (3.56–4.03)
* P*-trend	< 0.001	0.011	< 0.001	< 0.001	< 0.001	< 0.001
**Sex**						
Men	Reference					
Women	0.01 (0.01–0.01)	0.07 (0.06–0.07)	0.74 (0.72–0.76)	0.76 (0.74–0.78)	1.60 (1.55–1.66)	0.81 (0.79–0.84)
**Education**						
< High school	Reference					
≥ High school	1.06 (1.02–1.09)	1.24 (1.17–1.32)	0.84 (0.82–0.87)	0.63 (0.61–0.65)	0.77 (0.75–0.80)	0.75 (0.72–0.78)
**Annual household income (RMB)**						
< 50 000	Reference					
≥ 50 000	1.11 (1.08–1.15)	1.22 (1.15–1.29)	1.07 (1.04–1.10)	0.85 (0.83–0.87)	1.08 (1.05–1.12)	0.94 (0.91–0.97)
**Residence urbanity**						
Rural	Reference					
Urban	0.87 (0.84–0.90)	1.02 (0.96–1.07)	0.97 (0.95–0.99)	1.10 (1.07–1.13)	1.17 (1.13–1.21)	0.90 (0.87–0.93)
**Occupation**						
Non-farmer	Reference					
Farmer	1.20 (1.14–1.26)	0.96 (0.88–1.05)	0.85 (0.82–0.88)	1.34 (1.29–1.40)	0.81 (0.77–0.86)	1.13 (1.07–1.19)

**Table 4:** Adjusted Association between Age, Sex and Socioeconomic Status with Cardiovascular Risk Factor

	Smoking	Drinking	Overweight/obesity	Hypertension	Dyslipidemia	Diabetes
	**Adjusted Odds ratio (95% confidence interval)**					
**Age (years)**						
35–44	Reference					
45–54	0.97 (0.92–1.02)	1.18 (1.09–1.28)	1.32 (1.28–1.37)	2.31 (2.21–2.41)	2.02 (1.91–2.14)	1.97 (1.85–2.10)
55–64	1.14 (1.08–1.21)	1.24 (1.14–1.35)	1.26 (1.21–1.30)	4.35 (4.16–4.54)	3.20 (3.03–3.38)	3.09 (2.91–3.29)
65–75	0.82 (0.77–0.87)	1.06 (0.97–1.17)	1.07 (1.03–1.12)	6.74 (6.42–7.06)	3.41 (3.22–3.62)	3.70 (3.47–3.94)
* P*-trend	< 0.001	0.120	< 0.001	< 0.001	< 0.001	< 0.001
**Sex**						
Men	Reference					
Women	0.01 (0.01–0.01)	0.07 (0.06–0.07)	0.71 (0.70–0.73)	0.73 (0.71–0.75)	1.63 (1.57–1.69)	0.81 (0.78–0.83)
**Education**						
< High school	Reference					
≥ High school	0.61 (0.58–0.64)	0.92 (0.86–0.97)	0.81 (0.79–0.84)	0.83 (0.80–0.85)	-	0.94 (0.90–0.98)
**Annual household income (RMB)**						
< 50 000	Reference					
≥ 50 000	-	1.13 (1.07–1.20)	1.08 (1.06–1.11)	-	1.21 (1.17–1.25)	-
**Residence urbanity**						
Rural	Reference					
Urban	0.91 (0.87–0.95)	-	-	1.13 (1.10–1.16)	1.17 (1.13–1.21)	0.90 (0.87–0.93)
**Occupation**						
Non-farmer	Reference					
Farmer	-	-	-	-	-	-

Adjusted for age, sex, education, annual household income, residence urbanity, health insurance status, occupation and marital status

### Combination of CV risk factors

As shown in Table 5, compared to other CV risk factors, presence of overweight/obesity was more likely to coexist with other CV risk factors, including smoking (8.8%), hypertension (24.2%), dyslipidemia (10.0%) and diabetes mellitus (10.0%).

**Table 5:** Combination of CV Risk Factor

	Smoking	Drinking	Overweight/obesity	Hypertension	Dyslipidemia	Diabetes
**Smoking**	17.2%	3.1%	8.8%	7.2%	2.6%	3.0%
**Drinking**	-	5.3%	2.8%	2.5%	0.9%	1.0%
**Overweight/obesity**	-	-	48.9%	24.2%	10.0%	10.0%
**Hypertension**	-	-	-	39.9%	9.4%	9.5%
**Dyslipidemia**	-	-	-	-	18.6%	4.0%
**Diabetes**	-	-	-	-	-	16.1%

CV, cardiovascular

### Number of CV risk factors according to age, sex and SES

**Fig. 2:**
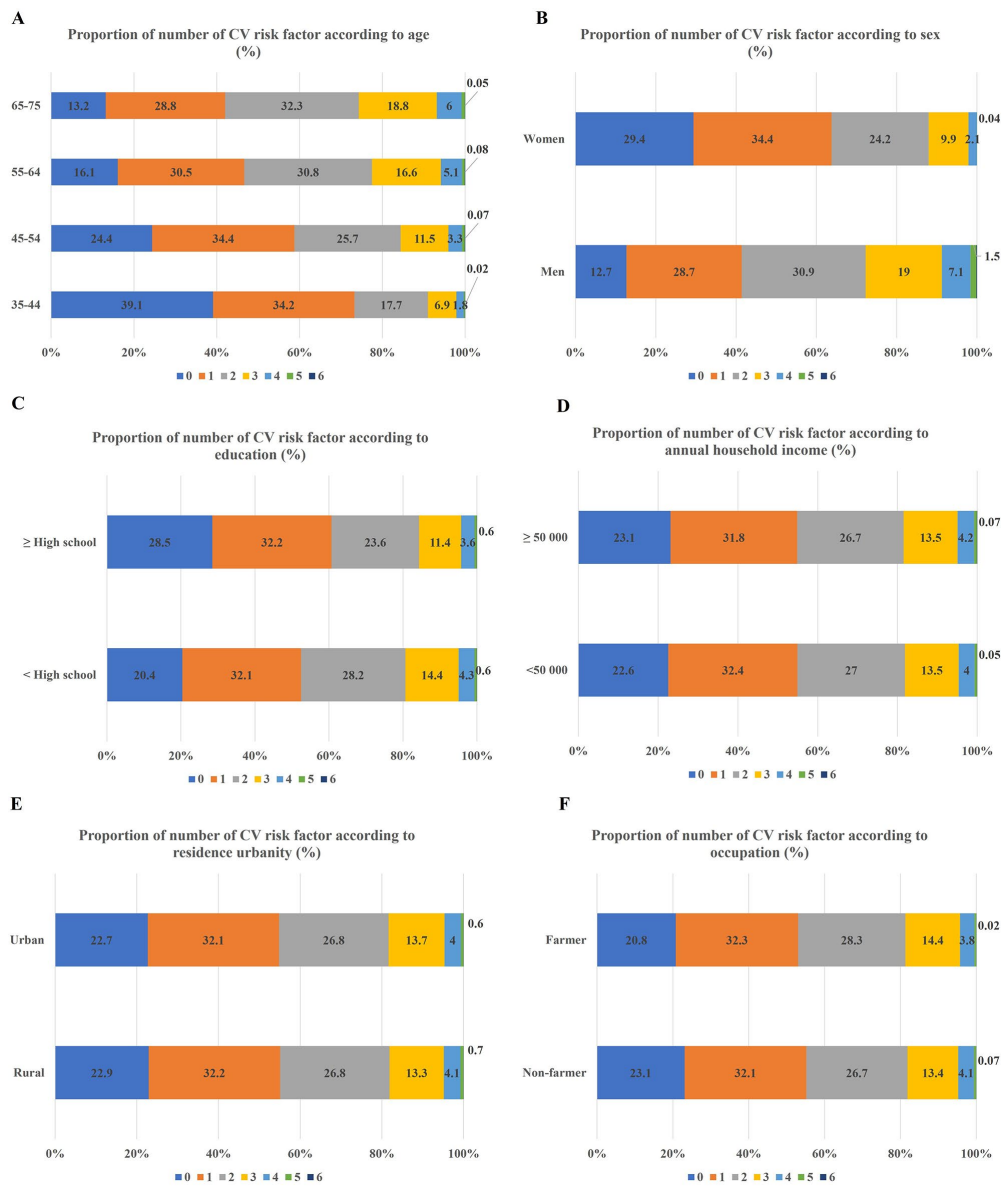
Number of cardiovascular risk factor according to sociodemographic status. Panel A: Proportion of number of cardiovascular risk factor according to age. The proportion of people with no risk factor decreased with age. Panel B: Proportion of number of cardiovascular risk factor according to sex. Women were more likely to have no risk factor than men. Panel C-F: Proportion of number of cardiovascular risk factor according to socioeconomic status. People with ≥ high school degree were more likely to have no risk factor. There was no statistically significant difference in the proportion of number of risk factor according to annual household income and residence urbanity. Farmer were less likely to have no risk factor

The proportion of participants with no risk factor decreased with increasing age groups (35–44 years 39.1% vs. 45–54 years 24.4% vs. 55–64 years 16.1% vs. 65–75 years 13.2%; P < 0.001; Fig. 2). In people aged 35–44, the proportion of those with at least 1 risk factor was more than 60%. Women were more likely than men to have no risk factor (29.4% vs. 12.7%). People with ≥ high school degree were more likely than those with < high school degree to have no risk factor (28.5% vs. 20.4%). Farmers were less likely than non-farmers to have no risk factor (20.8% vs. 23.1%).

## Discussion

To our knowledge, this is the first study to report the contemporary CV risk factors burden and its association with SDS in the community populations of Guangdong Province. There are some important findings. First, overweight/obesity and hypertension have become the two most common CV risk factors in Guangdong Province. Furthermore, overweight/obesity often coexists with other CV risk factors. Second, the burden of CV risk factors is high even in young people and increased with increasing age. Third, there are statistically significant differences in the burden of CV risk factors by sex, with women having lower burden than men. Fourth, the burden of CV risk factors varied substantially by SES. These findings provide scientific foundations for the allocations of health resources to prevent and manage these conventional CV risk factors at the population level, which may ultimately help reduce the CVD burden in the future.

## CV risk factors burden in guangdong province and other regions of China

Compared to the nationwide population of the China PEACE Million Persons Project [12, 13, 15, 16], the prevalence of smoking (20.0% vs. 17.2%), drinking (7.8% vs. 5.3%), overweight/obesity (53.5% vs. 48.9%), hypertension (44.7% vs. 39.9%) and dyslipidemia (33.8% vs. 18.6%) seemed to be numerically lower in Guangdong Province. These differences could be partly explained by the differences in SDS between Guangdong Province and other regions of China. Prior study suggests that in most provinces of China, the prevalence of CV risk factors were higher in urban than rural areas, which was probably due to higher levels of unhealthy lifestyles in rural residents [25]. Guangdong is one of the most developed provinces in China and participants in this study were from the most developed areas of Guangdong Province. It is thus probable that participants of the study had a relatively higher level of healthy lifestyles. Furthermore, people with well economic status are more likely to have regular health examination and seek medical services for health promotion [26]. In addition, people in developed areas are more likely to have high education attainment, which could facilitate them uptake health literacy. Indeed, in this study, we noticed that higher education attainment was associated with significantly lower odds of CV risk factors. Although having a lower prevalence of overweight/obesity, the prevalence of diabetes mellitus was numerically higher in Guangdong Province than the other two nationwide population-based studies (16.1% vs. 12.8% vs. 10.9%) [19, 20]. We are unsure the reasons. While these findings suggest a high burden of diabetes mellitus in Guangdong Province, and specific efforts are needed to reduce the burden. For example, public health promotion in terms of encouraging people to eat low-carbohydrate diet, do physical activity regularly, reduce sedentary time and decrease consumption of sugar-sweetened beverage could help to ease the burden of diabetes mellitus [10]. Overall, our results are consistent with prior nationwide population-based report that the burden of CV risk factors was lower in South China [12].

## High burden of overweight/obesity and hypertension in guangdong province

Among the six CV risk factors, overweight/obesity and hypertension are the two most common risk factors in the community populations of Guangdong Province. Given the high prevalence of these two risk factors, population-based cost-effective strategies are immediately needed. Some interventions have been demonstrated effective. For example, long-term modest salt reduction is associated with a significant reduction in blood pressure [27]. Unfortunately, one population-based survey showed that the average salt-intake for individual person was 7.6 g/day in Guangdong Province [28], which is higher than the World Health Organization recommendation (< 5 g/day). Labeling the adverse effects of high-salt intake in the container could be a cost-effective way to reduce salt in cooking. In addition, incentivizing the manufactures to use potassium chloride as a partial substitute for sodium chloride could have fundamental impacts on reducing salt-intake at the population level. We can also leverage the social media such as the WeChat or Weibo to advocate low salt diet, regular physical activity, and reduced energy-dense food consumption. Through these platforms, health knowledge can be disseminated rapidly and immensely. Furthermore, convenient apps such as the Apple Fitness Plus can help people do physical activity in a more scientific and effective way, and people can also use the apps to communicate with their counterparts, creating a positive feedback loop. Furthermore, algorithms derived from machine learning could be used to help better quantify the CV risk factors burden in these community populations. Taken together, we believe that these novel health-techs will be the solution to a lot of the cardiovascular challenges. Through combination of these health-techs with behavioral analysis, we can find solutions quicker and maintain the positive feedback loop between healthcare workers and the community populations.

We found that overweight/obesity often coexists with other risk factors, suggesting that overweight/obesity might play a central role during other CV risk factors development. Indeed, prior studies have demonstrated that overweight/obesity is significantly associated with elevated blood pressure [29], cholesterol and triglyceride [30] and blood glucose [31]. Therefore, preventing overweight/obesity development could concurrently help reduce the burden of hypertension, dyslipidemia and diabetes mellitus.

## Targeted populations and preventive strategies

Even in people aged 35–44, nearly 60% had at least 1 CV risk factor, and the burden of CV risk factors increased with increasing age, underscoring the importance of preventing CV risk factors development from young age. As overweight/obesity and hypertension are the two most common risk factors in young adults, regular moderate-to-vigorous physical activity combined with low-carbohydrate and low-salt diet could be a cost-effective way to prevent these risk factors development.

In general, women had a lower burden of CV risk factors than men, which is consistent with prior findings in a nationwide population-based study [25], suggesting that specific efforts would be needed to mitigate the CV risk factors burden in men. There were some sex-differences in the prevalence of risk factors. Compared to men, women were more likely to have dyslipidemia while less likely to have other risk factors. Among the nationwide population of the China-PEACE Million Persons Project, women were less likely to have overweight/obesity [13] but more likely to have dyslipidemia [16]. A lower prevalence of diabetes mellitus in women was observed in the other nationwide population-based study [19]. In a national level, women were more likely to have hypertension [15], which was contrast to our current study. These findings demonstrate the heterogeneous features of CV risk factors burden by sex.

There are variations in the association between individual SES metric and the burden of CV risk factors, indicating the complex relationship between SES and cardiovascular health. Notably, the association between education attainment and the burden of CV risk factors was consistent. Higher education attainment was associated with significantly lower odds of CV risk factors. Similar trends were also observed in the nationwide population of China PEACE Million Persons Project [13, 15, 16], demonstrating the importance of education in mitigating the burden of CV risk factors and reinforcing the necessary to promote education at the population level. Indeed, prior report demonstrates that lower education attainment is the second modifiable risk factor for CVD in middle-income country like China [32]. Higher annual household income was associated with higher odds of drinking, overweight/obesity and dyslipidemia, which might be due to adverse effects of nutritional excess. Indeed, in the context of rapid economic and cultural change, nutritional transition in Guangdong Province has followed the patterns that developed countries have experienced in the past. For example, people in this region are in favor of eating refined grains, edible oils and animal-source foods which is accompanied by substantial cooking behavior changes (e.g., frying and broiling with cooking oil) [33], leading to overweight/obesity and dyslipidemia. The association between urban residence and CV risk factor varied, which might be due to differential changes in economy and culture between the urban and rural areas. These findings suggest that specific prevention strategies are needed in the rural and urban areas. After adjusting for covariates, occupation was not associated with CV risk factors, suggesting that occupation per se might not be an independent determine of CV risk factors burden. In light of the variations of the CV risk factors burden by SES, it is needed to allocate health resources in a targeted way. For example, to individuals with low education level, we can use cardiovascular health videos to educate them on how to maintain healthy lifestyle; to individuals with low annual household income, implementation of public fitness equipment is crucial which can facilitate them to participate in regular physical activity; and to individuals who live in the rural areas, improvement in accessibility to the high quality of healthcare is fundamentally important.

## Limitations

There are some limitations of the study. First, the current study did not use random sampling method to recruit participants because it was not possible with such rapid, large-scale recruitment. This limitation might lead to overrepresentation of women and prohibited estimation of weighted prevalence of CV risk factor in Guangdong Province. Future studies using stratified multistage random sampling method are needed to obtain a representative sample of the general Chinese population. Second, participants in the study were from developed areas of Guangdong Province. Due to the urban-rural disparities in CV risk factor burdens, it is possible that the current study underestimated the burden of CV risk factors in Guangdong Province. It is needed to randomly sample participants from rural and urban areas in the future. Third, data on other CV risk factors such as daily salt-intake and physical inactivity were not captured. This limitation could have prevented us to investigate the reasons for a high prevalence of hypertension and overweight/obesity in Guangdong Province, which should be investigated in the future. Fourth, CV risk factors such as smoking and drinking were determined based on self-report, which was subject to recall biases and/or social stigma. Quantitative analysis of lifestyle habits could be analyzed with the help of the tech apps which can track intake, and if anonymized at source this may help overcome limitations due to recall bias and stigma. In addition, we assessed smoking and drinking status qualitatively. Further studies are needed to assess cigarette smoking and alcohol drinking in a quantitative way, which would provide more detailed information regarding the burden of these two risk factors at the population level.

## Conclusion

Findings of this study suggest that the burden of CV risk factors is high in Guangdong Province, and the burden is varied by SDS. Effective and targeted interventions are needed to reduce the burden of CV risk factors in Guangdong Province.

## Data Availability

The datasets generated and/or analysed during the current study are not publicly available as the current study is ongoing but are available from the corresponding author on reasonable request.

## Abbreviations

CV: Cardiovascular
SDS: Sociodemographic status
SES: Socioeconomic status
CVD: Cardiovascular disease
